# M2 Microglial Extracellular Vesicles Attenuated Blood-brain Barrier Disruption *via* MiR-23a-5p in Cerebral Ischemic Mice

**DOI:** 10.14336/AD.2023.0714

**Published:** 2024-05-07

**Authors:** Jia-Ji Pan, Lin Qi, Liping Wang, Chang Liu, Yaying Song, Muyassar Mamtilahun, Xiaowen Hu, Yongfang Li, Xiao Chen, Haroon Khan, Qun Xu, Yongting Wang, Yaohui Tang, Guo-Yuan Yang, Zhijun Zhang

**Affiliations:** ^1^Department of Neurosurgery, Huashan Hospital, Fudan University, Shanghai 200040, China.; ^2^School of Biomedical Engineering, Shanghai Jiao Tong University, Shanghai 200030, China.; ^3^Department of Neurology, Renji Hospital, School of Medicine, Shanghai Jiao Tong University, Shanghai 200120, China.; ^4^Department of Neurology, Ruijin Hospital, School of Medicine, Shanghai Jiao Tong University, Shanghai 200025, China.; ^5^Department of Neurosurgery, Ruijin Hospital, School of Medicine, Shanghai Jiao Tong University, Shanghai 200025, China.; ^6^Health Management Center, Department of Neurology, Renji Hospital of Medical School of Shanghai Jiaotong University, Shanghai, 200127, China

**Keywords:** blood-brain barrier, extracellular vesicles, microglia, miR-23a-5p, stroke

## Abstract

Protecting the integrity of the blood-brain barrier (BBB) is crucial for maintaining brain homeostasis after ischemic stroke. Previous studies showed that M2 microglial extracellular vesicles (EVs) played a neuroprotective role in cerebral ischemia. However, the role of M2 microglial EVs in maintaining BBB integrity is unclear. Therefore, we explored the mechanisms of M2 microglial EVs in regulating BBB integrity. To identify microglial EVs, we used nanoparticle tracking analysis, transmission electron microscopy, and western blot analysis. Adult male ICR mice were subjected to 90-min middle cerebral artery occlusion (MCAO), followed by the injection of PKH26-labeled M2 microglial EVs via the tail vein. After MCAO, we assessed brain infarct and edema volume, as well as modified neurological severity score. BBB integrity was measured by assessing IgG leakage. The effects of M2 microglial EVs on astrocytes and endothelial cells were also examined. To investigate the molecular mechanisms, we performed RNA sequencing, miR-23a-5p knockdown, and luciferase reporter assays. Our results showed that PKH26-labeled microglial EVs were mainly taken up by neurons and glial cells. M2 microglial EVs treatment decreased brain infarct and edema volume, modified neurological severity score, and IgG leakage, while increasing the ZO-1, occludin, and claudin-5 expression after MCAO. Knockdown of miR-23a-5p reversed these effects. RNA sequencing revealed that the TNF, MMP3 and NFκB signaling pathway involved in regulating BBB integrity. Luciferase reporter assay showed that miR-23a-5p could bind to the 3’ UTR of TNF. M2 microglial EVs-derived miR-23a-5p decreased TNF, MMP3 and NFκB p65 expression in astrocytes after oxygen-glucose deprivation, thereby increasing ZO-1 and Claudin-5 expression in bEnd.3 cells. In conclusion, our findings demonstrated that M2 microglial EVs attenuated BBB disruption after cerebral ischemia by delivering miR-23a-5p, which targeted TNF and regulated MMP3 and NFκB p65 expression.

## INTRODUCTION

Ischemic stroke is a disease with high morbidity, high mortality, and high disability, which has been receiving more and more attention [1]. Thrombolysis and arterial thrombectomy therapies could save many acute ischemic stroke patients; however, ischemia-induced secondary brain injury could cause serious brain edema and neurological dysfunction in survivors [2]. Therefore, we focused on exploring a new therapeutic approach for ischemic stroke.

The blood-brain barrier (BBB) is a dynamic structure that consists of endothelial cells, pericytes, astrocyte end-feet, and the basement membrane. It regulates the exchange of substances between the blood and the brain [3, 4]. Maintaining BBB integrity is essential for brain homeostasis [5]. However, in the acute phase of ischemic stroke, the BBB is severely disrupted, leading to damage tight junctions and BBB leakage [6]. This damage allows blood components to enter the brain parenchyma, resulting in irreversible brain injury [7]. Therefore, protecting BBB integrity is a promising strategy for ischemic stroke therapy.

After ischemic stroke, microglia in the brain were activated and polarized into M1 or M2 phenotypes [8]. M1 microglia increased the expression of pro-inflammatory cytokines such as IL-β and TNFα, while M2 microglia were involved in tissue repair, phagocytosis of debris, and activation of neurotrophic pathways [3]. Extracellular vesicles (EVs) are small vesicles that contain various substances, including proteins, lipids, and nucleic acids [9]. EVs derived from IL4-stimulated microglia, also known as M2 microglia, have been shown neuroprotective role following middle cerebral artery occlusion (MCAO) mice [10-12]. This indicated that the M2 microglial EVs could be a promising therapeutic approach for ischemic stroke. To comprehensively understand the role of M2 microglial EVs in ischemic stroke, we investigated whether M2 microglial EVs protect BBB integrity in MCAO mice and explored the underlying mechanisms.

In this study, we detected the BBB integrity in M2 microglial EVs-treated mice 3 days after MCAO. We found that M2 microglial EVs treatment increased the expression of tight junction proteins in brain endothelial cells. When we injected PKH26-labeled microglial EVs into the tail vein, they were primarily taken up by Tuj-1^+^ neurons, Iba-1^+^ microglia, and GFAP^+^ astrocytes. However, only a small number of PKH26-labeled microglial EVs were taken up by CD31^+^ endothelial cells, demonstrating that the high expression of tight junction proteins by M2 microglial EVs could not occur directly through endothelial cells. Further analysis using bulk RNA sequencing revealed that MMP3 was the most downregulated gene in M2 microglial EVs-treated mice compared to the PBS-treated mice. Since MMP3 is primarily expressed in astrocytes [13], we hypothesized that M2 microglial EVs could regulate the expression of tight junction proteins in endothelial cells by decreasing MMP3 expression in astrocytes. To test this hypothesis, we conducted *in vitro* assays using primary astrocytes and bEnd.3 cells. Previous study has shown that miR-23a-5p is highly enriched in M2 microglial EVs compared to M0 microglial EVs [12], indicating that it may play an essential role in the function of M2 microglial EVs. However, the role of miR-23a-5p in regulating the expression of tight junction proteins is still unclear. Therefore, we performed miR-23a-5p knockdown experiments to validate its role in regulating BBB integrity. Our study demonstrated that M2 microglial EVs could attenuate BBB disruption in MCAO mice by delivering miR-23a-5p, which targeted TNF and regulated MMP3 and NFκB p65 expression.

## MATERIALS AND METHODS

### Animal Experimental Design

The animal experimental procedures were approved by the Institutional Animal Care and Use Committee (IACUC) of Shanghai Jiao Tong University, Shanghai, China, and were conducted in accordance with the US National Research Council’s Guide for the Care and Use of Laboratory Animals. The animal studies were reported following the ARRIVE guidelines. Adult male Institute of Cancer Research mice (2 months old; Jiesijie, Shanghai, China) weighing 25 to 30 grams were used in this study. To investigate the role of M2 microglial EVs in BBB protection after MCAO, twenty-six mice were randomly divided into PBS and M2 groups. The mice were injected with PBS (200 μl/d) or M2 microglial EVs (100 μg in 200 μl/d) for 2 d after MCAO. To clarify whether M2 microglial EVs attenuated BBB disruption via miR-23a-5p, twenty-seven mice were randomly divided into the NC, M2 EVs, and k/d groups. The mice received MCAO surgery 3 d after stereotactic injection of negative control (NC) antagomiR or miR-23a-5p antagomir. Then, the mice in the NC, M2 EVs, and k/d groups received PBS (200 μl/d) or M2 microglial EVs (100 μg in 200 μl/d) for 2 d after MCAO. The mice were sacrificed 3 d after MCAO for further detection.

### M2 Microglia Culture

BV2 microglia cells were purchased from Shanghai Zhong Qiao Xin Zhou Biotechnology Company (Zhong Qiao Xin Zhou, Shanghai, China). The cells were grown in Dulbecco’s Modified Eagle’s Medium (DMEM, #11965126, Gibco) supplemented with 10% EVs-depleted FBS (#10099141, Gibco), 100 units/ml penicillin, and 100 μg/ml streptomycin (#15-140-122, Gibco) at 37°C in a humidified incubator with 5% CO2 and 95% air. To eliminate EVs, the FBS was centrifuged at 100,000 g for 18 h and then incubated at 56°C for 30 min to inactivate. The BV2 microglia were stimulated with 20 ng/ml IL-4 (#CK74, Novoprotein) for 48 h, and the cell culture supernatants were collected for EVs isolation [10].

### M2 Microglial EVs Isolation, Analysis, and Labeling

Differential ultracentrifugation was performed to isolate and purify EVs, as previously described [14]. Briefly, the supernatants from M2 microglia culture were first centrifuged at 300 g for 10 min, followed by centrifugation at 2000 g for 10 min to remove cells and dead cells. Subsequently, the remaining supernatants were centrifuged at 10,000 g for 30 min to remove debris. The final supernatants were ultracentrifuged at 100,000 g for 70 min to obtain the EVs. To eliminate contaminating proteins, the EVs were washed once with PBS and subjected to ultracentrifugation at 100,000 g for 70 min. Finally, the EVs were resuspended in PBS and stored at -80°C. The protein concentration of the EVs was determined using a BCA protein kit (#ZJ102, Epizyme). The particle diameter and concentration of EVs were assessed using Nanoparticle Tracking Analysis and Transmission Electron Microscopy. To label the EVs, the PKH26 Red Fluorescent Cell Linker Kit (#PKH26GL-1KT, Sigma-Aldrich) was utilized with minor modifications [15]. In brief, EVs and 2 μl of PKH26 dye were added to 500 μl of Diluent C, and the mixture was incubated for 4 min at room temperature in the dark. To eliminate excess dye, 200 μl of FBS was added, and the labeled EVs were washed with PBS at 100,000 g for 70 min. The EVs pellet was resuspended in PBS for further *in vitro* and *in vivo* assays.

### A Mouse Model of MCAO

The MCAO surgery was performed according to previously reported methods [10]. Briefly, mice were anesthetized using 1.2% isoflurane with a mixture of 60% nitrous oxide and 40% oxygen. The common carotid artery, external carotid artery, and internal carotid artery were isolated, and a silicone-coated 6-0 nylon suture was inserted into the internal carotid artery. The blood flow was then occluded for 90 min, followed by reperfusion. Cerebral blood flow was monitored using a laser Doppler perfusion and temperature monitor (Moor Instruments, Devon, UK). The criteria for confirming a successful MCAO model were a decrease in cerebral blood flow to 10% of the baseline level.

### Neurological Severity Assessment

The investigator blinded to the experiment performed the modified neurological severity score at 3 d after MCAO as previously reported [16]. Briefly, the investigator assessed the motor, sensory, reflex, and balance abilities of the mice and recorded the scores. A score of 1-6 indicated mild injury; 7-12 indicated moderate injury; 13-18 indicated severe injury.

### Brain Infarct Volume and Edema Measurements

Brain infarct and edema volume were measured following the previously described protocol [6]. A series of 20-μm-thick coronal brain slices were stained using cresyl violet (# MA0129, Meilunbio). The infarct volume was then measured using Image *J* software (National Institutes of Health, Bethesda, MD). The formula used for calculating the infarct volume was V = $\sum_{1}^{n}\left[\left(S_{n}+\sqrt[{}]{S_{n}*S_{n+1}}{+S}_{n+1}\right)\mathrm{*h}/3\right]$, where V represents the infarct volume, Sn indicates the infarct area, and h represents the distance between two adjacent brain slices. To calculate the edema ratio, the volume of the ipsilateral side was divided by the volume of the contralateral side.

### BBB Leakage Determination

To detect IgG leakage, brain slices were incubated in 0.3% hydrogen peroxide for 30 minutes, followed by serum blocking. After incubating the brain slices with biotinylated universal antibody (#PK-7200, Vector Laboratories) for 30 min, the slices were then incubated with DAB reagents (#SK-4105, Vector Laboratories) and hematoxylin (#C0105S, Beyotime Biotechnology). The integrity of tight junctions was assessed using double staining with ZO-1/CD31, Occludin/CD31, and Claudin-5/CD31. Brain sections were fixed by pre-cooled methyl alcohol for 10 min, followed by a 10-minute incubation with 0.3% Triton X-100. The slices were then blocked with 1% BSA for 1 h at room temperature. Subsequently, the brain slices were incubated overnight at 4°C with primary antibodies: anti-ZO-1 (1:200, #61-7300, Invitrogen), anti-Occludin (1:200, #33-1500, Invitrogen), anti-Claudin-5 (1:200, #35-2500, Invitrogen), anti-CD31 (1:200, #AF3628, R&D Systems), goat IgG isotype control (1:200, #02-6202, Invitrogen), rabbit IgG isotype control (1:200, #ab172730, Abcam), and mouse IgG isotype control (1:200, #MAB002, R&D Systems). After incubating the slices with fluorescence-conjugated secondary antibodies (1:500, #A21432, #A21206, #A21202, Invitrogen), they were mounted with coverslips for imaging using a confocal microscope (Leica, Solms, Germany). The leakage of IgG and the breakage of tight junction proteins were assessed using Image *J* software following previously described methods [6]. In each slice, four fields in peri-infarct areas were imaged, and a total of four slices were counted for each mouse brain. The quantification of gap formation for ZO-1 and occludin was calculated using the following formula: Gap formation (%) = (Gap length/Whole tight junction staining length) ×100%.

### RNA Sequencing (RNA-seq)

RNA-seq was performed as previously reported [6]. Mouse brain tissues in the peri-ischemic region were used for total RNA isolation using TRIzol reagent (#15596026, Thermo Scientific). The qualified RNA, with a RNA Integrity Number of ≥ 7, as evaluated by Agilent 2100 Bioanalyzer (Agilent Technologies, Santa Clara, CA), was used for further RNA-seq analysis. The libraries were constructed using the TruSeq Stranded mRNA LTSample Prep Kit (Illumina, San Diego, CA) for Illumina sequencing. Differential expression analysis and KEGG pathway enrichment analysis were performed between the PBS and M2 EVs groups.

### Real-time PCR Assay

Mouse brain tissues in the peri-ischemic region and primary astrocytes were collected to isolate total RNA using TRIzol reagent (Thermo Scientific). The cDNA was synthesized using a cDNA synthesis kit (#11123ES60, Yeasen) following the manufacturer’s protocol. The mRNA expression levels were then quantified using qPCR SYBR Green master mix (#11203ES08, Yeasen). The real-time PCR amplification parameters consisted of an initial denaturation step at 95°C for 30 s followed by 40 cycles of denaturation at 95°C for 5 s and annealing/extension at 60°C for 30 s. The expression level of mRNA was calculated using the 2^-ΔΔCt^ method and normalized to the expression of the endogenous control GAPDH [6]. The primers utilized in this study are detailed in Table 1.

**Table 1:** The primers used in the real-time PCR assay.

Gene name	Forward primers	Reverse primers
**TNF**	ATGTCTCAGCCTCTTCTCATTC	GCTTGTCACTCGAATTTTGAGA
**TLR4**	GCCATCATTATGAGTGCCAATT	AGGGATAAGAACGCTGAGAATT
**ICAM-1**	CTGAAAGATGAGCTCGAGAGTG	AAACGAATACACGGTGATGGTA
**MYD88**	CGGAACTTTTCGATGCCTTTAT	CACACACAACTTAAGCCGATAG
**RIPK1**	TACTTGCAAAGAAGAGTCGACT	ATAGGGTTCAGGTGTTCATCAG
**BLNK**	GAAGTGTTGTGGAAATCGTCAA	CACAGCATATTTCAGTCTCGTG
**TNFSF11**	GGAAGCGTACCTACAGACTATC	AAAGTGGAATTCAGAATTGCCC
**TRAF1**	CAAACATTGTTGCTGTCCTCAA	TCTTCCACAGGAAAGTACCATC
**CARD14**	GGTGAATGGCTCCTGCTACTTGTC	TCTCCGTCCCCTCCCCTCTG
**MMP2**	ACTTTGAGAAGGATGGCAAGTA	CTTCTTATCCCGGTCATAGTCC
**MMP3**	TGTCACTGGTACCAACCTATTC	TCTCAGGTTCCAGAGAGTTAGA
**MMP9**	CAAAGACCTGAAAACCTCCAAC	GACTGCTTCTCTCCCATCATC
**MMP10**	ACAAATGTGATCCTGCTTTGTC	ATCAAATGAAATTCAGGCTCGG
**MMP27**	CCCCAAATCCATCCACACACTCG	TCTGTCCATTGCTTGTGCCATCTC
**GAPDH**	GGTTGTCTCCTGCGACTTCA	TGGTCCAGGGTTTCTTACTCC

### Western Blot Analysis

Brain tissues in the peri-ischemic area and primary astrocyte were used for western blot analysis. The protein concentration was measured using a BCA protein kit (Epizyme). The proteins (30 μg/group) were separated into a 5-10% gradient SDS-PAGE gel and then transferred onto PVDF membranes. After blocking with 5% milk, the bands were incubated overnight at 4°Cwith primary antibodies, including anti-CD63 (1:1000, #sc-15363, Santa Cruz Biotechnology), anti-TSG101 (1:1000, #ab83, Abcam), anti-TNF (1:500, #sc-52746, Santa Cruz Biotechnology), anti-MMP3 (1:1000, # 17873-1-AP, ProteinTech Group), anti-NFκB p65 (1:1000, #8242T, Cell Signaling Technology), or anti-β-actin (1:1000, #66009, ProteinTech Group). Then the membranes were incubated with HRP-conjugated secondary antibody (1:5000, #HA1006 or #HA1001, HUABIO) at room temperature for 1 h. Visualization analysis was performed using an enhanced chemiluminescence substrate (#SQ201, Epizyme) and an imaging system (Bio-Rad, Hercules, CA).

### Primary Astrocyte Culture

Primary astrocytes were isolated from postnatal ICR mice (Jiesijie) using previously described methods [11]. The cortex was isolated and trypsinized at 37°C for 12 min. The cell suspension was filtered through 70-μm strainers (#CLS431751-50EA, Millipore). After centrifugation at 1000 rpm for 5 min, the cells were seeded onto a 6-well plate and cultured in an incubator at 37 °C with 5% CO2. After 24 h, the DMEM (Gibco) supplemented with 10% FBS (Gibco), 100 U/mL penicillin, and 100 mg/mL streptomycin (Gibco) was replaced, and the medium was subsequently replaced every 3 d.

### Oxygen-glucose Deprivation and Reoxygenation (OGD)

The OGD assay was conducted following the previously reported method [17]. The cell culture medium was substituted with glucose-free DMEM (#11966025, Gibco). Then the oxygen-glucose deprivation experiments were carried out in a chamber filled with a gas mixture containing 95% N2 and 5% CO2. The primary astrocytes were kept in the chamber for 5 h, while the bEnd.3 cells were exposed for 14 h. Afterward, the cells were removed from the chamber and the medium were replaced with DMEM supplemented with 10% FBS for a reoxygenation period of 3 h.

**Figure 1.**
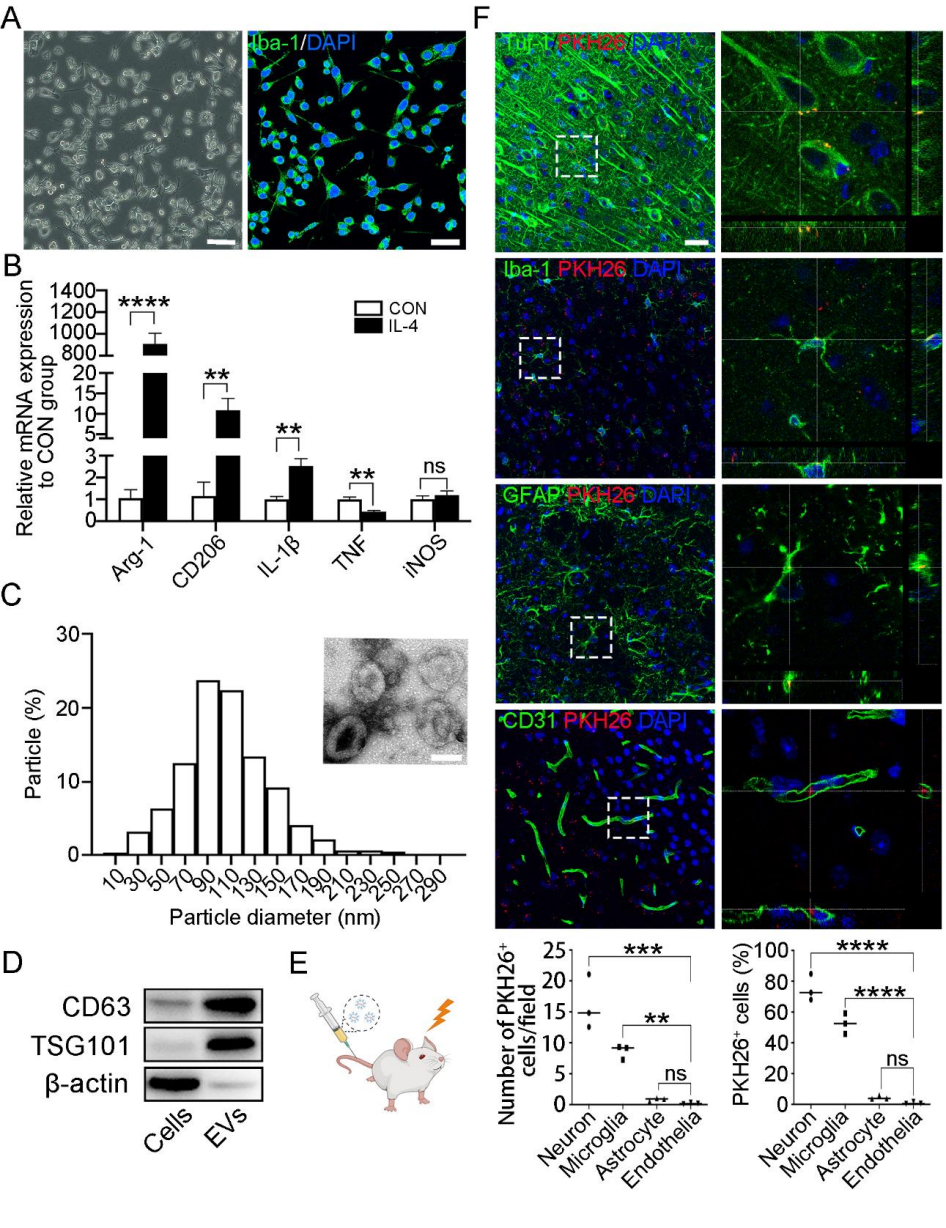
**Identification of extracellular vesicles (EVs) and EV distribution in mouse brains after cerebral ischemia**. (**A**) Representative images of microglia under the bright-field microscope and immunostaining of the microglia marker Iba-1. Scale bar, 100 (left)/50(right) μm. (**B**) Relative mRNA levels of Arg-1, CD206, IL-1β, TNF, and iNOS in control and IL-4 stimulated microglia. **P<0.01, ****P<0.0001 (n=3, unpaired two-tailed Student’s *t*-test). (**C**) Nanoparticle tracking analysis and the ultrastructural image showed the diameter of M2 microglial EVs. Scale bar, 100 nm. (**D**) Protein levels of TSG101, CD63, and β-actin in cells and EVs. (**E**) M2 microglial EVs were injected into middle cerebral artery occlusion (MCAO) mice via the tail vein. (**F**) Immunostaining of Tuj-1, Iba-1, GFAP, and CD31 in ischemic mouse brains after injection of PKH26-labeled M2 microglial EVs. Scale bar, 25 μm. Enlarged images showed the square area in the left images. The number of PKH26^+^ cells per field and the percentage of PKH26^+^ cells were measured (below). **P<0.01, ***P<0.001, ****P<0.0001 (n=3, one-way ANOVA with Tukey’s *post-hoc* test). ns indicates not significant; CON indicates control group; IL4 indicates IL4 stimulated group.

### Stereotactic Injection of MiR-23a-5p AntagomiR

The stereotactic injection of miR-23a-5p antagomiR was carried out following the previously described method [1]. ICR mice under anesthesia were immobilized on a stereotactic apparatus (RWD Life Science, Shenzhen, China). Next, miR-23a-5p antagomiR (#miR30017019-4-5, RIBOBIO) or NC antagomiR (#miR3N0000001-4-5, RIBOBIO) was injected into the left striatum region at a 2 mm lateral to the sagittal suture and 3 mm beneath the dura, with a pumping rate of 0.2 μl/min using a pump (WPI, Sarasota, FL). After 3 d, these mice were utilized for MCAO surgery.

### Dual-luciferase Reporter Assay

The dual-luciferase reporter assay was performed following a previously described protocol [12]. Briefly, luciferase vectors containing wild-type or mutated binding sites of the TNF 3’-untranslated region (3’-UTR) were constructed by OBiO Technology in Shanghai, China. 293T cells were cultured in 96-well plates for 24 h and then transfected with luciferase vectors and miR-23a-5p or NC mimics. After 48 h of transfection, luciferase activities were measured using the Dual-Luciferase Reporter Assay System (#E1910, Promega). The relative luciferase activity was calculated as the ratio of firefly luciferase activity to renilla luciferase activity.

### Statistical Analysis

Statistical analyses were performed using GraphPad Prism 8. The Shapiro-Wilks normality test was conducted to assess the normal distribution of the data. For normally distributed data, we utilized an unpaired two-tailed Student *t*-test or one-way ANOVA followed by Newman-Keuls or Tukey’s post-hoc test. For non-normally distributed data, we replaced the *t*-test with the Mann-Whitney test and the one-way ANOVA with the Kruskal-Wallis test followed by Dunn’s multiple comparisons test. The data were presented as mean±SD. *P*<0.05 was considered statistically significant. The figures displayed representative images that accurately represented the average data of all samples. The *P* values were indicated as asterisks in the figures and presented as ranges in the figure legends.

### Schematics

The schematic cartoons in Figures 1E and 4B were created using BioRender.com.

## RESULTS

### Identification of EVs and EV distribution in mouse brains after cerebral ischemia

BV2 microglia were stained with anti-Iba-1 and DAPI (Fig. 1A). The results showed that all BV2 microglia were Iba-1^+^. After 24 h of IL-4 stimulation, we observed an upregulation of the mRNA expression levels of the M2 microglial markers Arg-1 and CD206, while the M1 microglial marker TNF was downregulated (Fig. 1B). Nanoparticle Tracking Analysis and ultrastructure imaging indicated that the diameter of M2 microglial EVs was mainly between 30-150 nm (Fig 1C). Western blot analysis revealed that M2 microglial EVs, isolated through differential ultracentrifugation, exhibited high expression of the EV markers TSG101 and CD63, but low expression of β-actin (Fig 1D).

PKH26-labeled M2 microglial EVs were injected into MCAO mice via the tail vein (Fig. 1E). Neurons, microglia, astrocytes, and endothelial cells were stained with anti-Tuj-1, anti-Iba-1, anti-GFAP, and anti-CD31, respectively. The results showed that PKH26-labeled M2 microglial EVs localized in Tuj-1^+^ neurons, Iba-1^+^ microglia, GFAP^+^ astrocytes, and CD31^+^ endothelial cells (Fig. 1F). The statistical results indicated that M2 microglial EVs were mainly taken up by Tuj-1^+^ neurons, Iba-1^+^ microglia, and GFAP^+^ astrocytes (Fig. 1F), indicating that M2 microglial EVs may regulate BBB integrity through astrocytes, which are critical components of the BBB.

**Figure 2.**
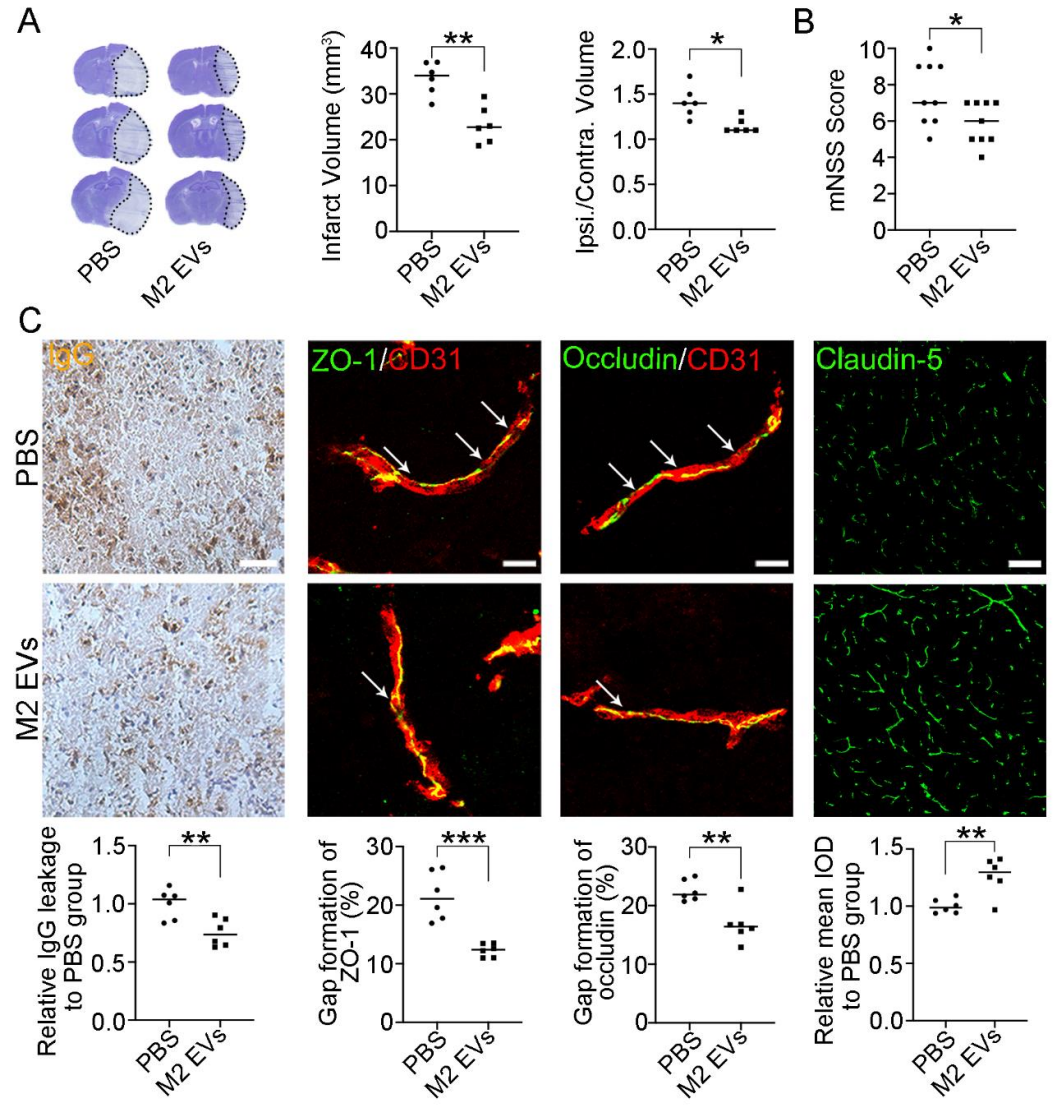
**M2 microglial EVs attenuated brain injury and blood-brain barrier (BBB) disruption**. (**A**) Cresyl violet staining of coronal brain sections in PBS and M2 microglial EVs-treated mice at 3 d after MCAO (left). The dashed line areas indicate infarction. Infarct volume and edema volume were calculated from brain sections (right). *P<0.05, **P<0.01 (n=6, unpaired two-tailed Student’s *t*-test for statistical analysis of Infarct Volume, Mann-Whitney test for statistical analysis of Ipsi. /Contra. Volume). (**B**) The modified neurological severity score. *P<0.05 (n=9, Mann-Whitney test). (**C**) Immunostaining of IgG, ZO-1, occludin, claudin-5, and CD31 in the perifocal area at 3 d after MCAO. Arrows indicate gaps caused by the breakage of tight junction proteins. Scale bar (left to right), 25/10/10/100 μm. **P<0.01, ***P<0.001 (n=6, unpaired two-tailed Student’s *t*-test). PBS indicates PBS-treated mice; M2 EVs indicates M2 microglial EVs-treated mice; Contra indicates contralateral; Ipsi indicates ipsilateral.

### M2 microglial EVs attenuated brain injury and BBB disruption

Cresyl violet staining was performed on mouse coronal sections to evaluate neuronal injury. The infarct volume and edema volume decreased in the M2 microglial EVs-treated mice compared to the PBS-treated group after MCAO (Fig. 2A). Furthermore, modified neurological severity score decreased in the M2 EVs group compared to the PBS group at 3 d after MCAO (Fig. 2B). Additionally, IgG leakage and breakage of tight junction proteins (ZO-1, Occludin, and Claudin-5) decreased in the M2 EVs group compared to the PBS group at 3 d after MCAO (Fig. 2C). Therefore, treatment with M2 microglial EVs may be effective in attenuating BBB disruption in the acute stage of MCAO.

**Figure 3.**
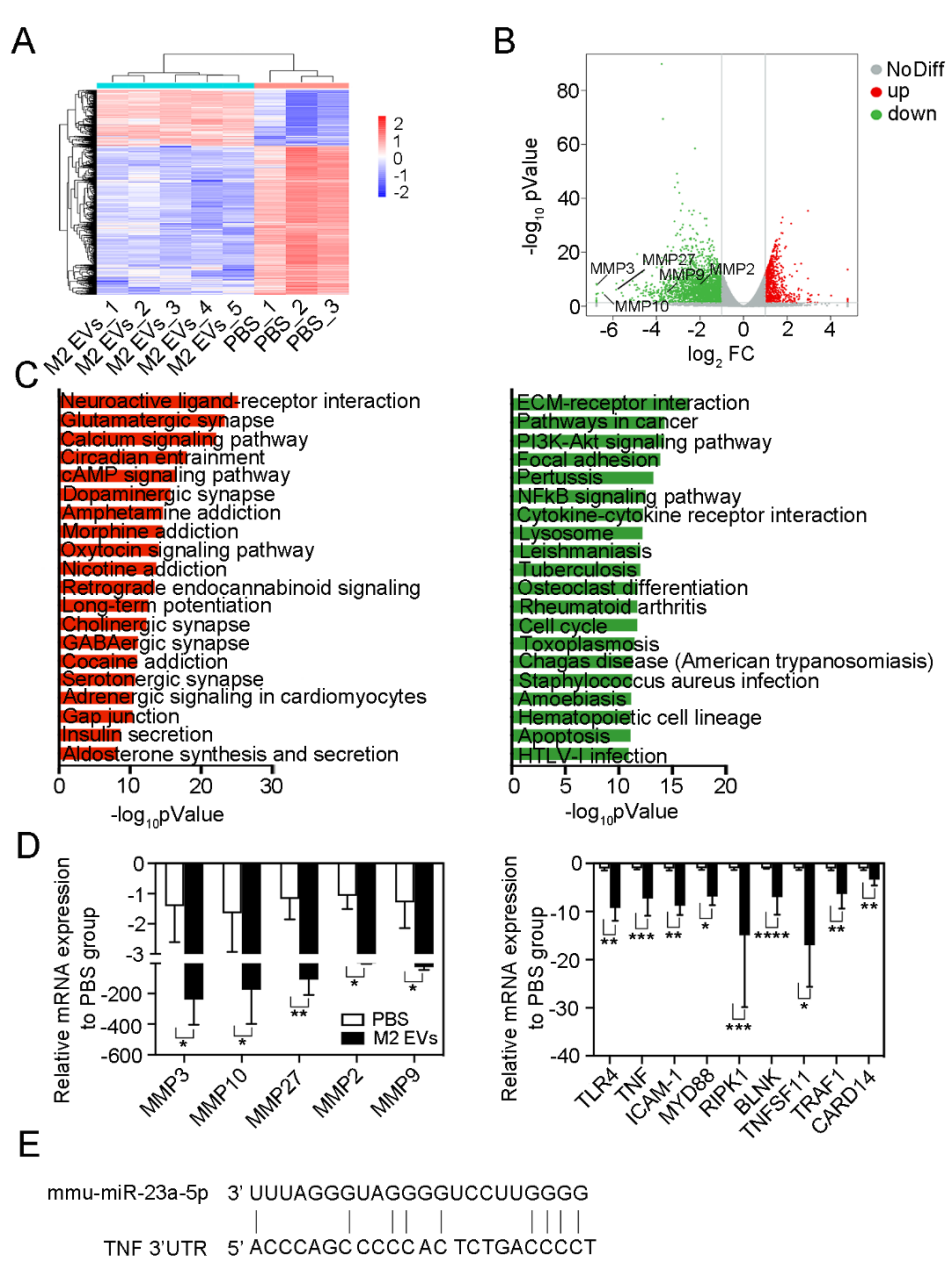
**RNA sequencing revealed underlying mechanisms of M2 microglial EVs in regulating BBB integrity**. The heatmap (A) and the volcano plot (B) show differentially expressed genes in the perifocal region at 3 d after MCAO. (**C**) KEGG pathway analysis of upregulated (red) genes and downregulated (green) genes. (**D**) Real-time PCR assay to validate several differentially expressed genes between the PBS (n=3) and M2 EVs (n=5) groups. *P<0.05, **P<0.01, ***P<0.001, ****P<0.0001 (Mann-Whitney test for statistical analysis of MMP9, MMP10, MYD88, and TNFSFSF11; unpaired two-tailed Student’s *t*-test for statistical analysis of other genes). (**E**) Predicted binding sites of miR-23a-5p in the 3’UTR of TNF. NoDiff indicates No difference; up indicates upregulated genes; down indicates downregulated genes; FC indicates fold change; 3’ UTR indicates 3’ untranslated region.

### RNA-seq revealed underlying mechanisms of M2 microglial EVs in regulating BBB integrity

Our previous work performed microRNA sequencing to identify differentially expressed microRNAs between M2 microglial EVs and M0 microglial EVs [12]. The results demonstrated that miR-23a-5p was the most upregulated miRNA in M2 microglial EVs compared to M0 microglial EVs, indicating its critical role in M2 microglial EVs [12]. However, it remains unclear whether M2 microglial EVs-derived miR-23a-5p regulates BBB integrity. To investigate the underlying mechanisms of M2 microglial EVs in BBB integrity regulation, we collected perifocal mouse brain tissues from PBS and M2 EVs group mice and performed RNA-seq analysis at 3d after MCAO. The heatmap displayed the differentially expressed genes between the M2 EVs group and PBS group at 3 d after MCAO (Fig. 3A). We identified a total of 3154 differentially expressed genes (foldchange>2, *P*<0.05), including 862 upregulated genes and 2292 downregulated genes (Table 1 in the Data Supplement). Among the downregulated genes, we identified MMP3 (foldchange=106), MMP10 (foldchange=89), and MMP27 (foldchange=58) as the top 3 downregulated genes (FPKM>2) marked in the volcano plot (Fig. 3B). Furthermore, the downregulated differentially expressed genes were enriched in inflammation-related signaling pathways, such as the NFκB signaling pathway (Fig. 3C). Real-time PCR results confirmed the RNA-seq findings, showing downregulation of matrix metalloproteinases (MMP3, MMP10, MMP27, MMP2, and MMP9) and molecules associated with the NFκB signaling pathway (TLR4, TNF, ICAM-1, MYD88, RIPK1, BLNK, TNFSF11, TRAF11, and CARD14) in the M2 EVs group compared to the PBS group (Fig.3D). Additionally, the miRWalk database predicted that miR-23a-5p could bind to the 3’UTR of TNF (Fig. 3E).

**Figure 4.**
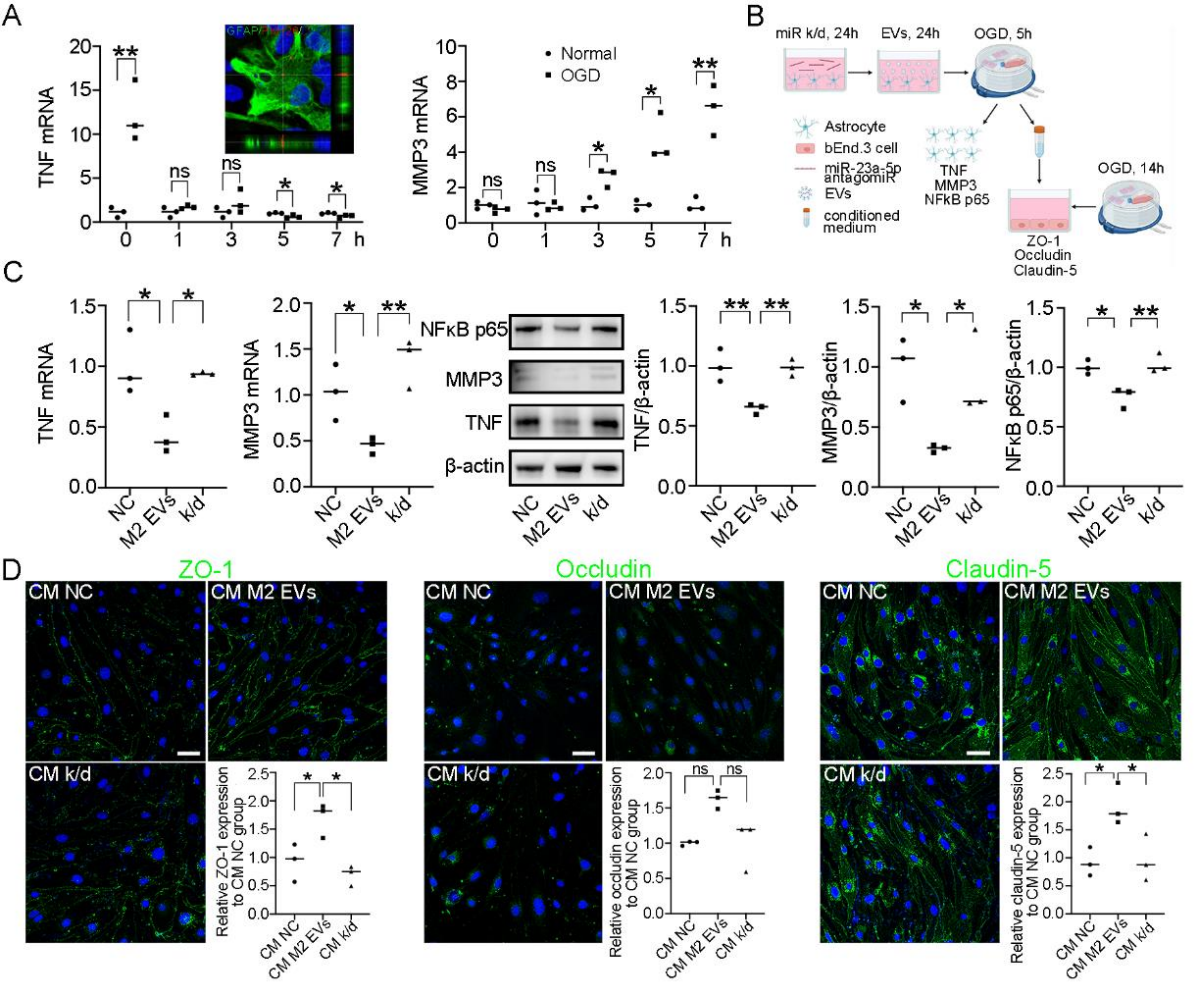
**M2 microglial EVs-derived miR-23a-5p downregulated TNF, MMP3 and NFκB p65 in astrocytes**. (**A**) Relative mRNA expression of TNF and MMP3 in primary astrocytes at 0, 1, 3, 5, and 7 h after OGD. The microscopic image shows the uptake of PKH26-labeled M2 microglial EVs in primary astrocytes. *P<0.05, **P<0.01 (n=3, unpaired two-tailed Student’s *t*-test or Mann-Whitney test). (**B**) Schematic diagram of *in vitro* assay. (**C**) Relative mRNA and protein expression of TNF, MMP3, and NFκB p65 in astrocytes after treatment with M2 Microglial EVs and miR-23a-5p antagomiR. *P<0.05, **P<0.01 (n=3, one-way ANOVA followed by the Tukey’s *post-hoc* test for statistical analysis of TNF/β-actin and NFκB p65/β-actin; one-way ANOVA followed by Newman-Keuls multiple comparisons test for statistical analysis of TNF mRNA, MMP3 mRNA, and MMP3/β-actin). (**D**) Immunostaining of ZO-1, Occludin, and Claudin-5 in bEnd.3 cells after treatment with conditioned medium from astrocytes. Scale bar, 50 μm. *P<0.05 (n=3, one-way ANOVA followed by the Tukey’s *post-hoc* test for statistical analysis of ZO-1 and claudin-5; Kruskal-Wallis test followed by Dunn’s multiple comparisons test for statistical analysis of occludin). ns indicates not significant; NC indicates negative control antagomiR-treated astrocytes; M2 EVs indicates M2 microglial EVs-treated astrocytes; k/d indicates miR-23a-5p antagomiR and M2 microglial EVs-treated astrocytes. CM NC indicates bEnd.3 cells treated with NC group astrocytes-derived conditioned medium; CM M2 EVs indicates bEnd.3 cells treated with M2 EVs group astrocytes-derived conditioned medium; CM k/d indicates bEnd.3 cells treated with M2 EVs group astrocytes-derived conditioned medium.

**Figure 5.**
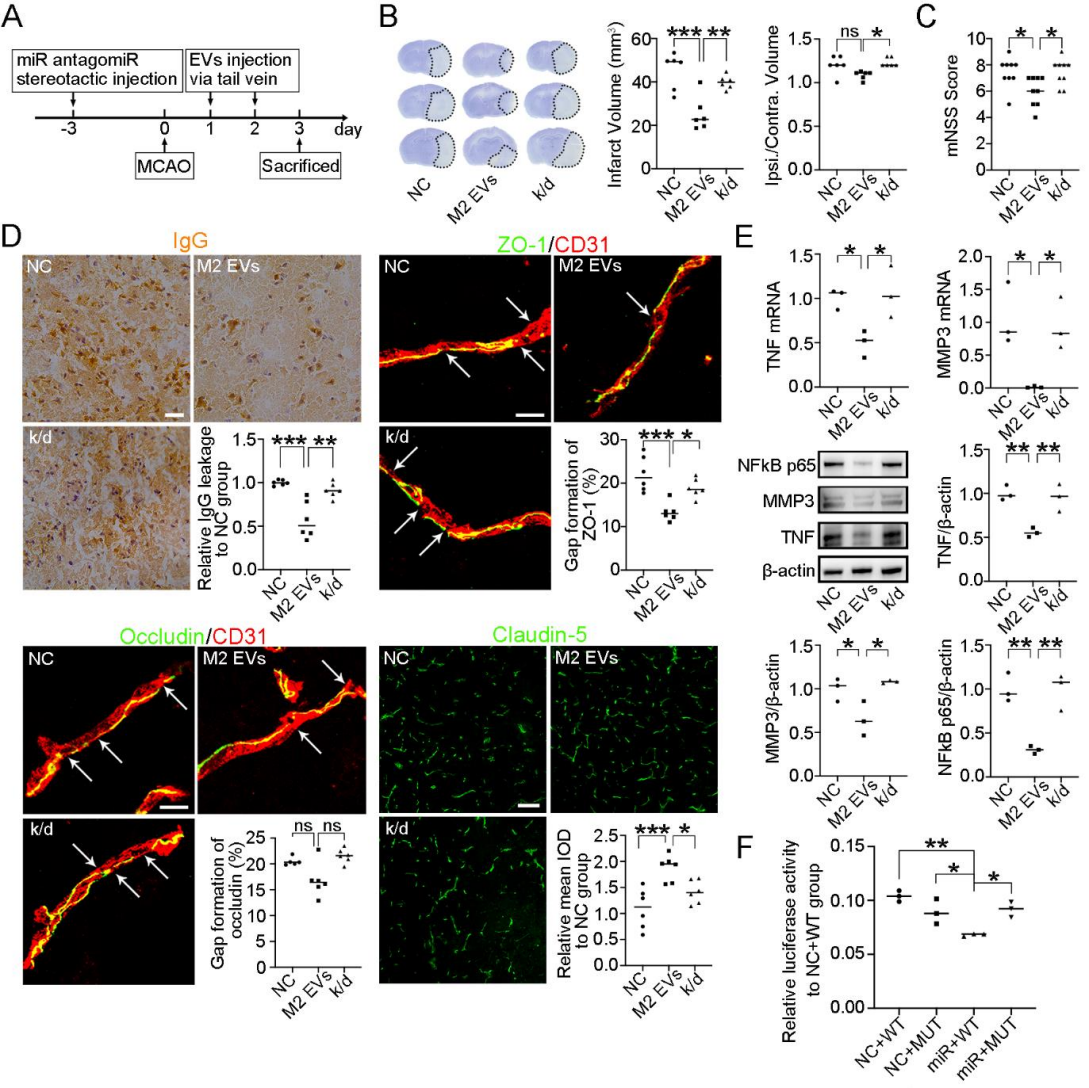
**M2 microglial EVs-derived miR-23a-5p attenuated BBB disruption and downregulated TNF, MMP3 and NFκB p65 in MCAO mice**. (**A**) Schematic diagram of the *in vivo* assay. (**B**) Cresyl violet staining of mouse brain sections at 3 d after MCAO. *P<0.05, **P<0.01, ***P<0.001 (n=6, one-way ANOVA followed by the Tukey’s *post-hoc* test for statistical analysis of Infarct Volume; Kruskal-Wallis test followed by Dunn’s multiple comparisons test for statistical analysis of Ipsi. /Contra. Volume). (**C**) The modified neurological severity score. *P<0.05 (n=9, one-way ANOVA followed by the Tukey’s *post-hoc* test). (**D**) Immunostaining of IgG, ZO-1, Occludin, Claudin-5, and CD31 in the perifocal area at 3 d after MCAO. Scale bar (left to right, top to bottom), 25/10/10/100 μm. *P<0.05, **P<0.01, ***P<0.001 (n=6, one-way ANOVA followed by the Tukey’s *post-hoc* test for statistical analysis of IgG, ZO-1 and claudin-5; Kruskal-Wallis test followed by Dunn’s multiple comparisons test for statistical analysis of occludin). (**E**) Relative mRNA and protein expression of TNF, MMP3, and NFκB p65 in the perifocal region at 3 d after MCAO. *P<0.05, **P<0.01 (n=3, one-way ANOVA followed by Newman-Keuls multiple comparisons test for statistical analysis of TNF mRNA, MMP3 mRNA; one-way ANOVA followed by the Tukey’s *post-hoc* test for statistical analysis of TNF/β-actin, MMP3/β-actin and NFκB p65/β-actin). (**F**) Dual-luciferase reporter assay showing miR-23a-5p could bind 3’-UTR of TNF. *P<0.05, **P<0.01 (n=6, one-way ANOVA followed by the Tukey’s *post-hoc* test). ns indicates not significant; NC indicates negative control antagomiR-treated mice; M2 EVs indicates M2 microglial EVs-treated mice; k/d indicates miR-23a-5p antagomiR and M2 microglial EVs-treated mice. NC+WT indicates negative control mimic+wild type 3’untranslated region; NC+MUT indicates negative control mimic+mutant 3’untranslated region; miR+WT indicates miR-23a-5p mimic+wild type 3’untranslated region; miR+MUT indicates miR-23a-5p mimic+mutant 3’untranslated region.

### M2 microglial EVs-derived miR-23a-5p downregulated TNF, MMP3 and NFκB p65 in astrocytes

The immunostaining image demonstrated that primary astrocytes were capable of internalizing PKH26-labeled M2 microglial EVs (Figure 4A). After subjecting astrocytes to oxygen-glucose deprivation for 5 h, the mRNA expression levels of TNF and MMP3 were assessed at 0, 1, 3, 5, and 7 h after oxygen-glucose deprivation. We found that TNF was initially upregulated at 0 h, but subsequently downregulated at 5 and 7 h, while MMP3 was upregulated at 3, 5, and 7 h (Fig. 4A). The experimental design was illustrated in a schematic diagram (Fig. 4B). To investigate the potential regulatory effects of M2 microglial EVs on TNF, MMP3, and NFκB p65 expression in astrocytes, primary astrocytes were isolated. Astrocytes were pretreated with M2 microglial EVs and miR-23a-5p antagomiR, followed by oxygen-glucose deprivation for 5 h and reoxygenation for 3 h. The results demonstrated that the mRNA and protein levels of TNF, MMP3, and NFκB p65 decreased in M2 microglial EVs-treated group compared to the NC group. However, miR-23a-5p knockdown reversed this effect (Fig. 4C). To investigate whether M2 microglial EVs could regulate the expression of endothelial tight junction proteins through astrocytes, the conditioned medium from NC, M2 EVs, and k/d group astrocytes was collected and used to treat bEnd.3 cells. Immunostaining results revealed that the conditioned medium derived from M2 EVs group astrocytes increased the expression of ZO-1 and Claudin-5 in bEnd.3 cells after 14 h of oxygen-glucose deprivation. Conversely, the conditioned medium from k/d group astrocytes reversed this protective effect (Fig. 4D).

### M2 microglial EVs-derived miR-23a-5p attenuated BBB disruption and downregulated TNF, MMP3 and NFκB p65 in MCAO mice

The schematic diagram illustrates the *in vivo* experimental design (Fig. 5A). Measurement of mouse coronal sections indicated that infarct volume decreased in the M2 EVs group compared to the NC group at 3 d after MCAO. However, miR-23a-5p knockdown reversed this effect (Fig. 5B). Additionally, the modified neurological severity score decreased in the M2 group compared to the NC group at 3 d after MCAO, but miR-23a-5p knockdown reversed this effect (Fig. 5C). In addition, miR-23a-5p knockdown reversed the protective role of M2 microglial EVs in maintaining BBB integrity, showing increased IgG leakage and disruption of tight junction proteins (ZO-1, Claudin-5, Fig. 5D). The mRNA and protein expression levels of TNF, MMP3, and NFκB p65 were downregulated in the M2 EVs group compared to the NC group, but miR-23a-5p knockdown reversed this effect (Fig. 5E). The dual-luciferase reporter gene assay showed that miR-23a-5p could bind to the 3’-UTR of TNF (Fig. 5F).

## DISCUSSION

Our study demonstrated that M2 microglial EVs could decrease the infarct and edema volume, ameliorate BBB disruption, and improve functional recovery 3 d after MCAO. The mechanistic study revealed that M2 microglial EVs attenuated BBB disruption in the acute stage of cerebral ischemia by delivering miR-23a-5p, which targeted TNF and regulated MMP3 and NFκB p65 expression.

EVs play a crucial role in cell-to-cell communication, and several studies have reported the essential role of EVs-derived microRNAs in this process [10, 18, 19]. In our previous study, we found that miR-23a-5p was the most abundant microRNA in M2 microglial EVs compared to the M0 microglial EVs. Furthermore, we confirmed that M2 microglial EVs-derived miR-23a-5p promoted white matter repair and functional recovery after cerebral ischemia in mice [12]. However, the role of miR-23a-5p in maintaining BBB integrity remains largely unknown. Our study demonstrated that M2 microglial EVs-derived miR-23a-5p improved BBB integrity by reducing IgG leakage and tight junction breakage in cerebral ischemic mice 3 d after MCAO. However, our study does not exclude the potential contribution of other miRNAs or proteins present in M2 microglial EVs to BBB integrity, which needs to be further investigated.

To identify the downstream targets of M2 microglial EVs, we conducted bulk RNA-seq in PBS and M2 microglial EVs-treated mice 3 d after MCAO. Bulk RNA-seq results showed several downregulated genes, with MMP3 being the most significantly downregulated gene among them. Real-time PCR analysis further confirmed the downregulation of MMP3 in M2 microglial EVs-treated mice compared to PBS-treated mice. MMPs are a group of proteolytic enzymes that are known to degrade extracellular matrix and tight junction proteins [20]. MMPs could exacerbate brain injury during the acute stage and promote recovery during the late stage [21]. MMP3 is one of the main MMPs inducible in the brain and activated MMP3 could directly activate MMP9 [22, 23]. Previous studies have shown that MMP3 contributes to the tPA-induced hemorrhagic transformation and worsens functional outcomes after hyperglycemic stroke, partially due to MMP3 degrading tight junction proteins [24]. Since MMP3 is mainly expressed in astrocytes [13], our *in vitro* results showed that M2 microglial EVs decreased the mRNA and protein levels of MMP3 in astrocytes after OGD. However, miR-23a-5p knockdown reversed this effect. MMP2, MMP3 and MMP9 are critical MMPs involved in BBB opening after ischemic stroke [25, 26]. Thus, we believed that M2 microglial EVs regulated BBB integrity through modulation of the multiple MMPs.

The miRWalk results indicated that miR-23a-5p did not have any predicted 3’ UTR binding sites of MMP3, suggesting that miR-23a-5p may indirectly regulate MMP3 expression and activity. To identify the direct target of miR-23a-5p, we analyzed the downregulated genes in the RNA-seq data and identified the enriched KEGG pathways. The results showed that the NFκB signaling pathway was one of the most relevant pathways in regulating BBB integrity. A previous study has reported that activation of the NFκB pathway upregulated pro-inflammatory factors, which can worsen brain injury after intracranial hemorrhage [27]. But downregulating NFκB has been shown to reduce brain edema and neurological dysfunction after brain ischemia/reperfusion [28]. We found that TNF and TLR4 as potential targets of miR-23a-5p in the NFκB signaling pathway. Furthermore, we found that TLR4 is a validated target of miR-23a-5p through dual-luciferase reporter assay [29]. Our study demonstrated that miR-23a-5p could bind to the 3’-UTR of TNF via dual-luciferase reporter assay. Moreover, we detected that M2 microglial EVs decreased the mRNA and protein levels of TNF in astrocytes and MCAO mice. But miR-23a-5p knockdown reversed this effect. Previous studies reported that TNF induced MMP3 in astrocytes and human cerebral endothelial cells [30, 31]. Hence, M2 microglia EVs-derived miR-23a-5p decreased MMP3 by inhibiting TNF in MCAO mice.

Although we demonstrated that the M2 microglial EV was a promising treatment for ischemic stroke, there were still some limitations in the study. Firstly, the EV isolation method should be improved to obtain purer and higher-yield EVs. The use of an iodixanol-based high-resolution density step gradient has been reported for the isolation of distinct EV species [32]. Secondly, nonspecific uptake of M2 microglial EVs allowed them to enter various brain cells and regulate brain function. However, non-targeted therapy caused unpredictable side effects. Thus, the M2 microglial EVs modification to achieve targeted therapy is the future direction for ischemic stroke therapy. Thirdly, a recent study recommended avoiding the use of M1 and M2 labels to investigate microglial function [33]. In our study, to simplify the microglia nomenclature, we referred to IL4-stimulated microglia as M2 microglia.
